# Mouthwash Use and the Risk of Oral, Pharyngeal, and Laryngeal Cancer. A Meta-Analysis

**DOI:** 10.3390/ijerph18158215

**Published:** 2021-08-03

**Authors:** Sorin Hostiuc, Ioana Victoria Ionescu, Eduard Drima

**Affiliations:** 1 Department of Legal Medicine and Bioethics, Faculty of Dental Medicine, Carol Davila University of Medicine and Pharmacy, 042122 Bucharest, Romania; 2 Faculty of Dental Medicine, Carol Davila University of Medicine and Pharmacy, 050474 Bucharest, Romania; ioana.ivey@gmail.com; 3 Clinical Medical Department, Faculty of Medicine and Pharmacy, Dunărea de Jos University, 800654 Galați, Romania; drima_eduardpolea@yahoo.com

**Keywords:** cancer, mouthwash, alcohol, acetaldehyde, oral

## Abstract

Objective: The main aim of this study was to test whether the use of mouthwash is associated with subtypes of squamous cell carcinoma of the head and neck (SCCHN) and to test the potential risk of SCCHN depending on the mouthwash use duration, frequency, or alcoholic content. Materials and methods: We performed a meta-analysis using Web of Science and Scopus databases to detect the risk change associated with mouthwash use depending on the alcohol content, duration and frequency of use, and anatomical location. We used a random-effects model with the Sidik–Jonkman estimator for effect size model measures. Results: We included 17 studies in the meta-analysis containing 17,085 cases and 20,032 controls. The risk difference for SCCHN between mouthwash users and non-users was minimal, with a value of −0.02 [−0.05, 0.01]. Alcoholic mouthwash use was associated with a minimal decrease in risk (of −0.01 [−0.07, 0.05]). Frequent usage of mouthwash was associated with a statistically significant risk increase for SCCHN but the risk increase was marginal (0.04, [0.01, 0.06]). Conclusions: Overall, our study failed to show a statistically significant correlation between mouthwash use and the risk of SCCHN. The only statistically significant correlation that we could identify was between frequent usage and SCCHN, potentially caused by prolonged contact between some constituents of mouthwash (possibly alcohol) and the oral epithelium.

## 1. Introduction

Squamous cell carcinoma of the head and neck (SCCHN—which includes neoplasms of the oral cavity, pharynx, and larynx) is one of the most common tumor types, causing significant morbidity and mortality, especially in the Middle East, Asia, and Africa [1,2].

The most important risk factors for SCCHN, namely tobacco, alcohol, and HPV infection [3], can be controlled—through lifestyle choices for the first two and vaccination for the latter [4]. Other risk factors include infections with Candida albicans [5], Epstein–Barr virus [6], and ionizing radiation [7], but also some appertaining specifically to oral health, such as continued oral trauma, poor oral hygiene, the frequency of brushing, and (possibly) the use of mouthwash [8].

Mouthwashes are liquid antiseptic solutions that decrease the microbial load in the oral cavity, although there are unique formulations for other uses as well, including analgesic, anti-inflammatory, anti-fibrinolytic, antifungal, or cosmetic. Alcohol is often used in mouthwashes for its antiseptic functions and as a carrier for some active ingredients, including menthol or thymol, helping them penetrate the plaque [9,10]. The carcinogenic effects of alcohol in oral cancers are known. They are generated through multiple mechanisms, in which alcohol is involved either directly or through acetaldehyde, formed in the oral cavity through the microbial catabolism of ethanol. Moreover, as acetaldehyde cannot be further catabolized in the oral cavity, its levels are 10–100-fold higher than blood levels [11]. Ethanol leads directly to increased carcinogenesis in the oral cavity by inducing DNS hypomethylation—which alters the expression of proto- and anti-oncogenes, decreases the metabolism of CYP2E1, and subsequently decreases the levels of retinoic acid [12], leading to immune deficiency that promotes oncogenesis [13], and by acting as a carrier for other carcinogens (such as those found in tobacco or betel) [1,14]. Increased acetaldehyde levels cause disruptions of DNA synthesis and repair and the formation of stable DNA adducts—the binding of acetaldehyde to various proteins leads to structural and functional alterations of enzymes involved in DNA repair and methylation, and of proteins with anti-oxidative functions, such as glutathione [12]. Mouthwashes contain various other chemical agents that may cause irritation of the oral mucosa, including sweeteners and artificial colorings [15]. 

A potential association between the usage of mouthwashes and an increased risk of oral cancer was initially hypothesized by Weaver et al. in 1979 [16], who showed that from 200 patients with SCCHN, only eleven abstained from alcoholic beverages and tobacco; from those eleven, ten were heavy mouthwash users for more than 20 years and used brands containing more than 25% alcohol [16].

Further studies evaluating this association had contradictory results, with some supporting and others contradicting it. Gandini et al., in 2012, performed a meta-analysis that showed no statistically significant association between mouthwash use and the risk of oral cancer [17]. Aceves Argemi et al. recently completed an updated meta-analysis, also failing to show a potential association between mouthwash use and an increased risk of oral cancer [15]. Many articles evaluating potential connections between mouthwash and SCCHN involved more granular analyses, considering the potential relationship between mouthwash use and the type of SCCHN or examining the risk of developing an SCCHN depending on the duration of use, the number of times per day the mouthwash was used, or its alcoholic content, as, even if overall the risk of SCCHN is not associated with mouthwash use, there might still be a clinically significant correlation in one or more subgroups. This study aimed to test whether mouthwash use is associated with a subtype of SCCHN and to investigate the potential risk of SCCHN depending on the mouthwash use duration and frequency, and alcoholic content.

## 2. Materials and Methods

This meta-analysis was performed based upon the Preferred Reporting Items for Systematic Reviews and Meta-Analyses (PRISMA) and Meta-Analysis of Observational Studies in Epidemiology (MOOSE) guidelines [18,19].

### 2.1. Search Method

We analyzed articles extracted from Scopus and Web of Science (all databases) from the first year of each database up to February 2021 using the following keywords: “mouthwash” AND “oral cancer,” “mouthwash” AND “head cancer,” “mouthwash” AND “pharyngeal cancer”, and “mouthwash” AND “laryngeal cancer”. To add data from gray literature, we searched on all databases in Web of Science (allowing us to cover high-quality conference papers), analyzed books by using the Google Books website (which allows in-document searches), and performed searches with the same keywords in ResearchGate and Google Scholar. We downloaded relevant articles, checked their references, and downloaded additional articles that we considered appropriate from the reference list. All downloaded articles were included in a Paperpile database.

Two authors performed each search; the differences were noted, and a third reviewer reevaluated the studies for which differences were found when comparing the two lists before including them in the final analysis.

### 2.2. Selection Criteria

We used the following inclusion criteria: (1) studies containing information about the numbers of cases and controls that were mouthwash users/non-users, or which had data that allowed us to determine these values; (2) studies published in English and present in major online databases. The following exclusion criteria were employed: (1) the absence of relevant data; (2) case reports or case series; (3) less than 100 cases. Cases were considered subjects with SCCHN and controls were subjects without SCCHN.

### 2.3. Data Collection and Analysis

For each study, two reviewers extracted the data separately and included it in an Excel 365 database. A third reviewer combined the databases and checked the results for inconsistencies. We used the following information: study name, country of origin, type of study, inclusion and exclusion criteria; we also included the number of cases and controls, use of mouthwash in cases and controls—overall, gender-based (if given), the alcohol content of the mouthwash, usage interval (short/long term), frequency (less/more than once daily)—and the location of cancer—oral, pharyngeal, or laryngeal. We considered high alcohol-content mouthwashes to contain over 25% alcohol and low alcohol-content mouthwashes to contain less than 25% alcohol (as per the initial study performed on this topic [17]).

### 2.4. Quality Assessment and Risk of Bias

We used the Newcastle Ottawa Scale for case-control studies, which yields scores from 0 to 9, to assess the quality of the articles included in our analysis [20].

### 2.5. Statistical Analysis

We used Jamovi and Microsoft Excel 365 for macOS for statistical analyses. We employed a random-effects model with the Sidik–Jonkman estimator for effect size model measures [21]. We used the funnel plot and Egger’s regression test for plot asymmetry to analyze publication bias. I^2^ was used to test the presence heterogeneity between studies with the following thresholds: 0–35%—most likely not significant, 36–55%—moderate heterogeneity, 56–85%—most likely substantial heterogeneity, and 86–100%—significant heterogeneity (average values, based on [22]). We used 95% for confidence intervals (CI). Statistical significance was set at a *p* < 0.05.

## 3. Results

### 3.1. Search Synthesis

Initially, we obtained 1001 articles in Scopus and 606 in Web of Science. After removing duplicates and reviewing the abstracts, we selected 56 to be further analyzed. We added one other article from the other resources mentioned above.

**Figure 1:**
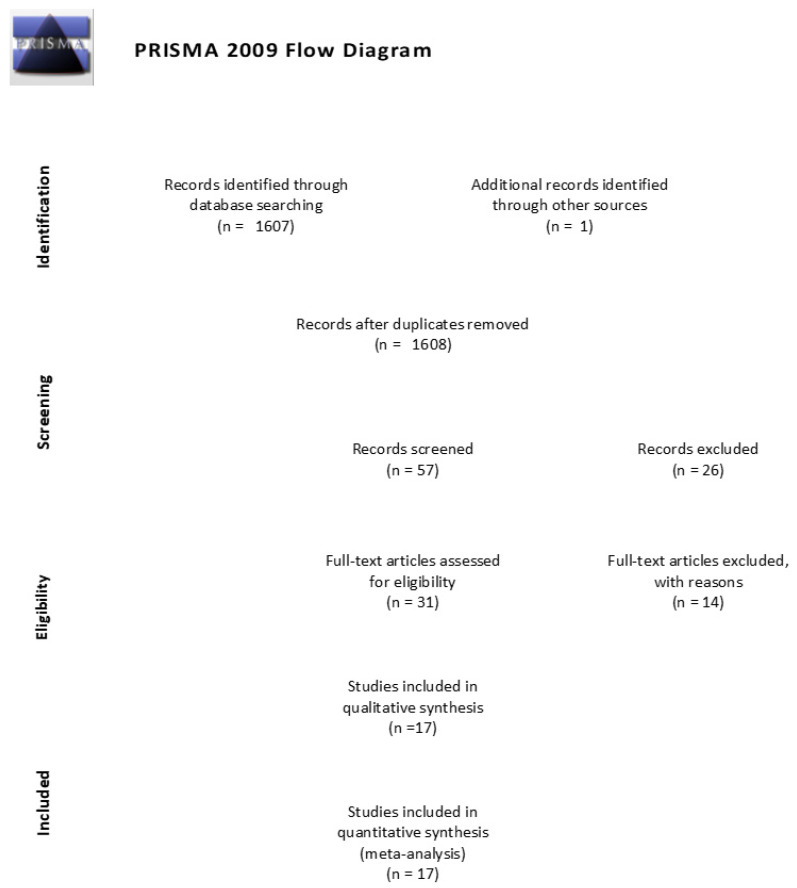
PRISMA flow diagram [23].

From the 57 articles, 17 were finally included in the meta-analysis. Fifteen studies were identified by both reviewers and two additional ones by reviewer 2 (HS). The inter-evaluation similarity was 88%. Details about the search synthesis are presented in Figure 1 [19]. We detail the papers contained in the meta-analysis in Table 1.

### 3.2. Quality Assessment

The included studies had quality scores ranging from 3 to 8 out of 9, with an average value of 6.24. The quality score for each study is given in Table 1.

### 3.3. The Overall Risk of Upper Aerodigestive Tract Cancers Associated with Mouthwash Use

**Figure 2:**
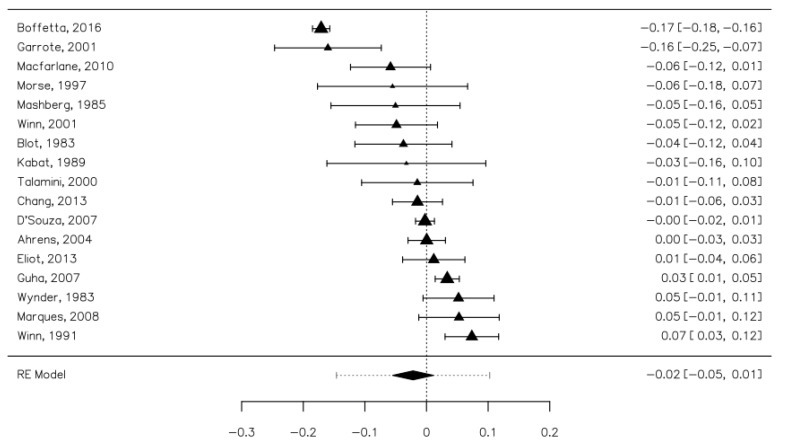
Forrest plot. The overall risk of upper aerodigestive tract cancers associated with mouthwash use.

We included 17 studies in the analysis, containing 17,085 cases and 20,032 controls. Overall, mouthwash usage was 37.88% in the cases group and 47.06% in the control group. The risk difference between the cases and controls was minimal at −0.02 [−0.05, 0.01] and not statistically significant (Figure 2). The heterogeneity of the studies was high (I^2^ = 92.07%). Publication bias was not statistically significant (Z = −0.472, *p* = 0.637). Five studies contained distinct datasets for women. In this group, the risk difference was 0.06 [−0.03, 0.16], which was also not statistically significant. The heterogeneity of the studies was lower (I^2^ = 75.86%). Three studies contained distinct datasets for men. In this group, the risk difference was 0.00 [−0.07, 0.06]. The heterogeneity of the studies was lower (I^2^ = 64.12%).

### 3.4. The Risk of Upper Aerodigestive Tract Cancers Associated with Mouthwash Depending on the Alcohol Content

In five studies, there was a clear separation in the results between users of alcoholic versus non-alcoholic/low-alcohol mouthwashes (less than 25%) [8,28,34,37,38]. The risk difference associated with the usage of alcoholic mouthwash use was minimal, with a value of −0.01 [−0.07, 0.05], which was not statistically significant. The heterogeneity of the studies was high (I^2^ = 84.25%). Publication bias was statistically significant (Z = −2.159, *p* = 0.031). Four studies contained specific data about the use of non-alcoholic/low-alcohol mouthwash [8,28,34,38]. The decrease in risk associated with the use of non-alcoholic/low-alcohol mouthwash between the cases and control groups was minimal, with a value of 0.00 [−0.03, 0.02], which was not statistically significant. The heterogeneity of the studies was average (I^2^ = 60.45%). Publication bias was not statistically significant (Z = 0.383, *p* = 0.702).

### 3.5. The Risk of Upper Aerodigestive Tract Cancers Associated with Mouthwash Depending on the Frequency and Duration of Use

Ten studies contained data that allowed us to establish the risk of SCCHN cancers associated with occasional mouthwash use (less than once a day) [24,26,28,30,31,32,33,35,37,39]. These studies contained 9604 cases, of which 2152 were occasional mouthwash users, and 11,355 controls, of which 2439 were occasional mouthwash users. The risk difference between the cases and control groups was minimal, with a value of 0.00 [−0.06, 0.07], which was not statistically significant. The heterogeneity of the studies was high (I^2^ = 97.22%). Publication bias was not statistically significant (Z = −0.625, *p* = 0.532).

**Figure 3:**
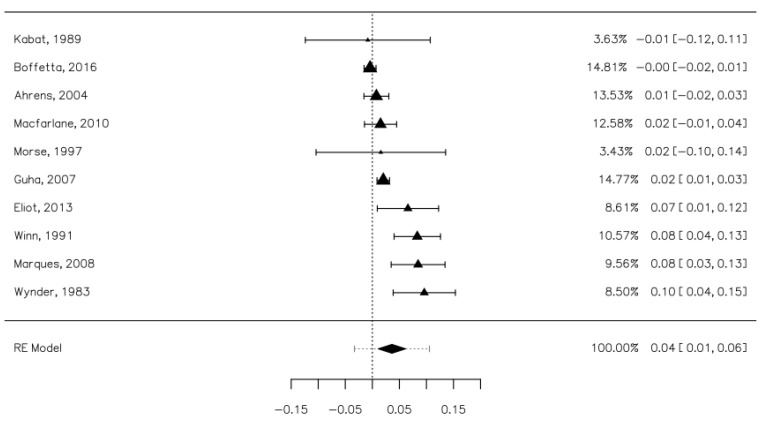
Forrest plot. The risk of upper aerodigestive tract cancers associated with frequent mouthwash use.

The same studies evaluated the risk of SCCHN associated with frequent usage (more than once a day). These studies included 10,750 cases (of which 1699 were mouthwash users) and 11,355 controls (1660 were mouthwash users). The risk associated with regular mouthwash use was significantly increased for the cases group, but the risk increase was marginal, specifically a 4% increase [0.01, 0.06] (Figure 3). The heterogeneity of the studies was high (I^2^ = 86.71%). Publication bias was not statistically significant (Z = 0.943, *p* = 0.346).

Seven studies included data allowing us to establish the risk of SCCHN associated with a shorter duration of mouthwash use [25,26,31,35,37,38,39]; they included 6825 cases, of which 1009 were occasional mouthwash users, and 8555 controls, of which 1377 were occasional mouthwash users. There was no risk difference between the cases and control groups, with the model yielding a value of 0.00 [−0.04, 0.04]. The heterogeneity of the studies was high (I^2^ = 82.97%). Publication bias was not statistically significant (Z = 0.541, *p* = 0.588).

The same studies evaluated the risk of SCCHN associated with more extended usage (the last category given by each study that analyzed the risk of SCCHN depending on the duration of mouthwash usage). These studies included 6825 cases (of which 1443 were mouthwash users) and 8555 controls (1634 were mouthwash users). The risk associated with regular mouthwash use was 0.01, with a 95%CI between −0.04 and 0.05. The heterogeneity of the studies was high (I^2^ = 81.99%). Publication bias was not statistically significant (Z = −1.806, *p* = 0.071).

### 3.6. The Risk of Oral, Pharyngeal, and Laryngeal Cancer Associated with Mouthwash Use

**Figure 4:**
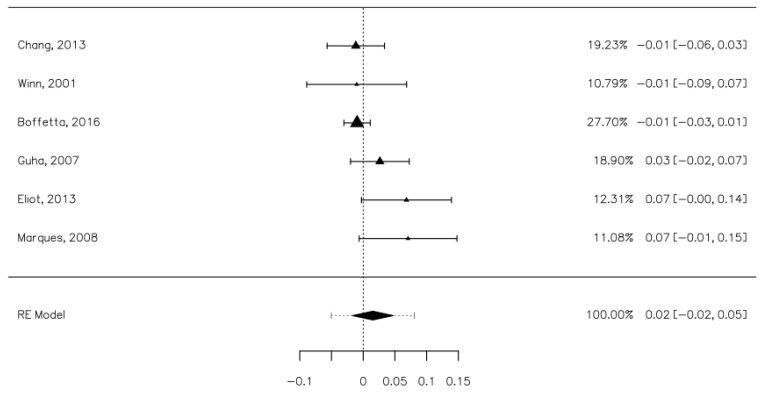
Forrest plot. The risk of oral cancer associated with mouthwash use.

Six studies contained data that allowed us to establish the risk of oral cancers associated with mouthwash use [8,26,28,30,33,38]. These studies included 3833 oral cancer participants, of which 1475 were mouthwash users and 13,018 controls, of which 5194 were mouthwash users. The risk difference between the cases and control groups was minimal at 0.02 [−0.02, 0.05], a value which was not statistically significant (Figure 4). The heterogeneity of the studies was moderate (I^2^ = 59.35%). Publication bias was not statistically significant (Z = −1.344, *p* = 0.179).

**Figure 5:**
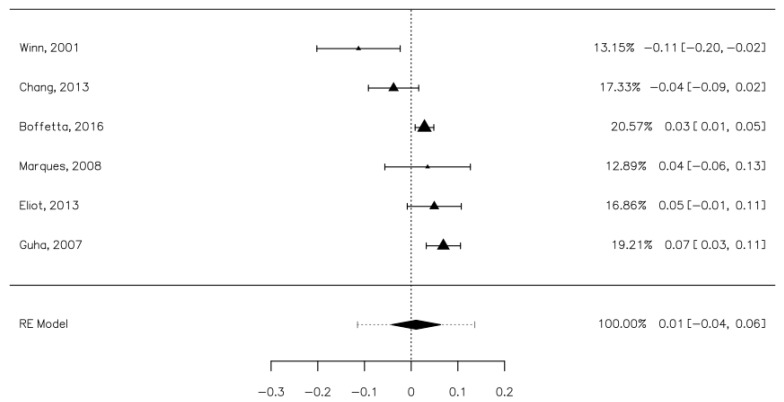
Forrest plot. The risk of pharyngeal cancer associated with mouthwash use.

Six studies contained data that allowed us to establish the risk of pharyngeal cancers associated with mouthwash use [8,26,28,30,33,38]. These studies included 4385 pharyngeal cancer participants, of which 1890 were mouthwash users, and 12,415 controls, of which 4930 were mouthwash users. The risk difference between the cases and control groups was minimal, with a value of 0.01 [−0.04, 0.06], which was not statistically significant (Figure 5). The heterogeneity of the studies was high (I^2^ = 86.33%). Publication bias was not statistically significant (Z = −1.122, *p* = 0.262).

**Figure 6:**
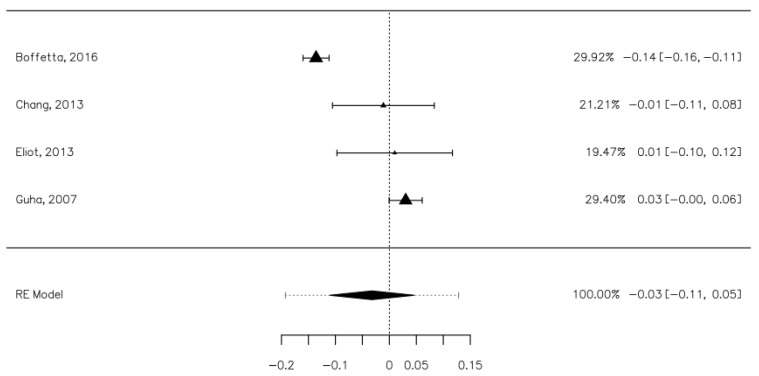
Forrest plot. The risk of oral laryngeal cancer associated with mouthwash use.

Four studies contained data that allowed us to establish the risk of laryngeal cancers associated with mouthwash use [8,26,28,30]. These studies included 2448 laryngeal cancer participants, of which 602 were mouthwash users, and 7984 controls, of which 2903 were mouthwash users. The risk difference between the cases and control groups was minimal, with a value of −0.03 [−0.11, 0.05], which was not statistically significant (Figure 6). The heterogeneity of the studies was average (I^2^ = 91.33%). Publication bias was not statistically significant (Z = 0.713, *p* = 0.476).

## 4. Discussion

Overall, our study failed to show a statistically significant correlation between mouthwash use and the risk of SCCHN. The risk difference between mouthwash users and non-users was minimal (−0.02, with a 95% confidence interval between −0.05 and 0.01), suggesting a minimal (and not statistically significant) protective effect of mouthwash use. Gender differences were also minimal, with a 6% risk increase for women and a 0% risk increase for men (both not statistically significant). The result was similar to other meta-analyses in the field: Aceves Argemi et al. found an OR of 1.057 (when not considering the presence of alcohol) [15], while Gandini et al. found an RR of 1.13 [17]. These results may have been caused by either an inconsistent pattern of mouthwash use (the usage of different brands with varying alcoholic content, varying temporal consumption trends, etc.), which cannot be easily quantified using retrospective studies, or by methodological inconsistencies, as the studies that were included in the analysis had different methodologies, used varying thresholds for establishing subgroups, etc. Better results could be obtained during prospective studies, with strictly controlled variables; however, such studies are difficult to design due to the relative rarity of SCCHNs and the need for an increased timespan to properly identify relevant trends.

In the next step of our analysis, we tested whether alcohol usage affects the SCCHN risk. Both subgroups (users of mouthwash with high and low/no alcohol) showed similar results, well within the margin of error. This result was similar to that of Aceves Argemi et al., who found an OR of 1.48 (also not statistically significant) for users of high-alcohol mouthwashes [15]. Duration of mouthwash use was not associated with significantly increased SCCHN risk. Still, frequent usage (more than once a day) led to a significant increase, albeit with marginal clinical importance (a 4% risk increase for regular users compared to the control group). This result contradicted the study by Gandini et al., who failed, using meta-regression, to find statistically significant differences between frequent and non-frequent mouthwash users [17]. As the most well-known carcinogen found in mouthwash is alcohol, and as most mouthwashes used to contain alcohol, it is possible that this result was caused by frequent usage of high-alcohol mouthwashes. High usage frequency, especially when associated with a non-watery rinse of the residual mouthwash, might lead to an increased contact time between alcohol and the epithelial tissue. We must also consider the fact that mouthwash (or even other potential carcinogens, such as tobacco) can be counterbalanced by the presence/usage of preventive factors. For example, a study performed in nine EU countries has shown that consuming fruits (at least once per day), vegetables excluding potatoes (at least once per day), or salads (at least once per day) significantly decreased the risk for SCCHNs after adjusting for gender, smoking, alcohol consumption, and education [32]. The association between frequent mouthwash usage should be further evaluated using studies specifically designed to test this hypothesis. We must also consider the possibility of this correlation being caused by a confounder, as mouthwash can be used to mask tobacco-related odors [17]. Also, taking into account the retrospective nature of most studies evaluating the potential correlation between mouthwash use and the risk of SCCHNs, which did not involve clear differentiation between non-alcoholic and alcoholic mouthwashes, and the varying ethanol concentrations between mouthwashes [25,31], it is difficult to properly assess the actual SCCHN risk increase associated with alcoholic mouthwashes.

The last part of our analysis tested whether mouthwash use can be explicitly linked to oral, pharyngeal, or laryngeal cancer. As their anatomical location predisposes tissues of the upper aerodigestive tract to numerous potential carcinogens, there is increased tissular vulnerability to cancers [40]. As discussed in the Introduction section, mouthwashes contain a few potential and proven carcinogens, of which the most discussed and analyzed is ethanol [9,11,41]. Our results showed no statistically significant association between mouthwash use and specific types of upper aerodigestive tract cancer, even though there was a minimal risk increase for oral cancer (where the contact between the mouthwash and epithelium is longer) and a minimal decrease for laryngeal cancer (where the direct contact between mouthwash and epithelium does not usually happen). This result, associated with a statistically increased risk connected to frequent usage, might imply a potential connection between direct, regular contact of the mouthwash and the oral epithelium. Still, more studies are needed to test this hypothesis specifically.

### Limitations

First, the results presented in various articles were inconstant, especially regarding subgroups (duration of use, frequency of mouthwash use, types of cancer included, etc.). This caused us to use approximations in determining which studies should be included in which group, leading to increased heterogeneity in the results. Second, we preferred to use the Sidik–Jonkman estimator, which produces more robust results than DerSimonian–Laird but tends to increase the confidence intervals and potentially misses some marginally statistically significant results. Third, SCCHNs are not the only types of cancers in this region. Even if some studies analyzed this type of cancer explicitly, others were not specifically focused on it or did not specify exactly what subtypes of cancer they had analyzed. Overall, our study failed to show a statistically significant correlation between mouthwash use and the risk of SCCHN. The only statistically significant correlation that we could identify was between frequent usage and SCCHN, potentially caused by prolonged contact between some constituents of mouthwash (possibly alcohol) and the oral epithelium. We preferred not to separate these types of studies, as most oral and neck cavity cancers were of the squamous cell carcinoma type.

## 5. Conclusions

Overall, our study failed to show a statistically significant correlation between mouthwash use and the risk of SCCHN. The only statistically significant correlation that we could identify was between frequent usage and SCCHN, potentially caused by prolonged contact between some constituents of mouthwash (possibly alcohol) and the oral epithelium. However, the results were inconclusive, as actual patterns of mouthwash use may be very inconsistent (usage of different brands with varying alcoholic content, varying temporal consumption trends, etc.) and cannot be easily quantified using retrospective studies. Better results could be obtained during prospective studies with strictly controlled variables; however, such studies are difficult to design due to the relative rarity of SCCHNs and the need for an increased timespan to properly identify relevant trends.

## Figures and Tables

**Table 1:** Studies included in the analysis.

*Study (Year)*	Country	Study Type	Timeframe	Cancer Type	Quality Score	Gender
Ahrens, 2014 [24]	Nine EU countries	Case-control	2002–2005	Oral, pharyngeal, laryngeal	6	Both
Blot, 1983 [25]	US	Case-control	1975–1978	Oral, pharyngeal	6	Female
Boffeta, 2016 [26]	Multiples sites	Case-control	Unspecified	Head and neck, oral cavity, pharyngeal, laryngeal	4	Both
Chang, 2013 [8]	Taiwan	Case-control	2010–2012	Oral, oropharynx, hypopharynx, larynx	8	Both
D’Souza, 2007 [27]	US	Case-control	2000–2005	Oral, pharyngeal	7	Both
Eliot, 2013 [28]	US	Case-control	2006–2011	Head and neck	7	Both
Garrote, 200 [29]	Cuba	Case-control	1996–1999	Oral, oropharynx	6	Both
Guha, 2007 [30]	Latin America, Eastern Europe	Case-control	1998–2003	Oral, pharyngeal	4	Both
Kabat, 1989 [31]	US	Case-control	1983–1987	Oral, pharyngeal	4	Female
Macfarlane, 2010 [32]	Europe	Case-control	Unspecified	Upper aerodigestive tract	7	Both
Marques, 2008 [33]	Brazil	Case-control	1998–2002	Oral, pharyngeal	7	Both
Mashberg, 1985 [34]	US	Case-control	1981–1983	Oral, pharyngeal	8	Both
Morse, 1997 [35]	US	Case-control	1990–1993	Oral epithelial dysplasia	6	Both
Talamini, 2000 [36]	Italy	Case-control	1996–1999	Oral, pharyngeal	3	Both
Winn, 1991 [37]	US	Case-control	1984–1985	Oral, pharyngeal	8	Both
Winn, 2001 [38]	US	Case-control	1992–1995	Oral, pharyngeal	8	Both
Wynder, 1983 [39]	US	Case-control	1977–1980	Oral, pharyngeal	7	Both
